# Embracing the Wisdom of Ancient Greece in the Era of Personalized Medicine—Uncertainty, Probabilistic Reasoning, and Democratic Consensus

**DOI:** 10.3389/ti.2023.12178

**Published:** 2023-10-26

**Authors:** Maarten Naesens

**Affiliations:** Department of Microbiology, Immunology and Transplantation, KU Leuven, Leuven, Belgium

**Keywords:** transplantation, biomarkers, prediction, precision medicine, artificial intelligence

## Abstract

Further improvements of outcome after solid organ transplantation will depend on our ability to integrate personalized medicine in clinical routine. Not only better risk stratification or improved diagnostics, also targeted therapies and predictive markers of treatment success are needed, as there is a virtual standstill in the development and implementation of novel therapies for prevention and treatment of allograft rejection. The integration of clinical decision support algorithms and novel biomarkers in clinical practice will require a different reasoning, embracing concepts of uncertainty and probabilistic thinking as the ground truth is often unknown and the tools imperfect. This is important for communication between healthcare professionals, but patients and their caregivers also need to be informed and educated about the levels of uncertainty inherent to personalized medicine. In the translation of research findings and personalized medicine to routine clinical care, it remains crucial to maintain global consensus on major aspects of clinical routine, to avoid further divergence between centres and countries in the standard of care. Such consensus can only be reached when experts with divergent opinions are willing to transcend their own convictions, understand that there is not one single truth, and thus are able to embrace a level of uncertainty.

## Introduction

In the closing plenary session of the 2023 Congress of the European Society for Organ Transplantation (ESOT) in Athens (17th September to 20th), I gave my impression of the past congress, building a bridge between the past, the present and the future of transplantation, drawing inspiration from the wisdom of ancient Greece and the earliest years of the democratic city-state of Athens 2,500 years ago.

I embarked on a journey into the world of transplantation 20 years ago. Those were the days of bustling international conferences. The field was vibrant, driven by collaborations between academia, clinical centres, and the pharmaceutical industry. New drugs emerged from decades of research, and clinical trials like the Symphony trial in kidney transplantation [1] shaped post-transplant patient management with effective immunosuppressive regimens that minimized the risk of acute rejection. The energy, innovation, and enthusiasm continue to inspire many of us working in transplantation today.

Fast forward to today, and the protocols established in the early 2000s remain largely unchanged. For instance, in the US, a country that usually embraces and implements innovation quickly, tacrolimus-mycophenolic acid is still the baseline immunosuppressive regimen for the vast majority of patients [2]. In another example, data from an ESOT survey presented at the ESOT Congress 2023 illustrates that the first-line treatment of T-cell-mediated rejection in kidney transplantation consists mainly of high-dose steroids, toxic therapy with numerous side effects. Second-line treatment, often needed as first-line therapy fails, consists of lymphocyte depleting antibodies, strong immunosuppressants that also have considerable additional risks; also these antibodies are in routine clinical use for 25 years. No new therapies are approved for the treatment of T cell-mediated rejection since several decades.

The clinical protocols developed several decades ago are largely unbeaten up to today, leaving us with the idea that we are playing extensions, no longer the real game. However, the field cannot relax given the very negative balance between moderate efficacy and long-lasting side effects, and excess mortality associated with the old-fashioned and limited therapeutic armamentarium we have available to prevent and treat rejection.

## Hippocrates and Personalized Transplant Medicine

In contrast to the lack of novel therapies entering our field and the protocolized care using standard regimens from 20–30 years ago, a remarkable transformation has taken place within the academic and research sphere—the advent of personalized medicine. The idea that “one size does not fit all” has never been more relevant. We hear this often, but the true meaning of it is underestimated. We must tailor our approach to individual patients, using tools to assess risk, monitor their health or disease, predict potential complications [3]. Notably, post-transplant care is not the only aspect that needs personalization; organ donor characteristics and organ quality vary significantly, impacting post-transplant outcomes and recipient wellbeing [4]. This need for personalization of our approaches was emphasized in many sessions at the ESOT Congress 2023.

Although one might think that this focus on personalized medicine is new, that is clearly not true. Already 2,500 years ago, Hippocrates, the father of medicine, noted that medicine is not absolute, thus its directions cannot be generalized to everybody [5]; that each human body/organism is different and responds differently to therapy, and therefore, the same treatment cannot be suitable for everybody; finally, that the physician should choose the appropriate treatment, depending on the patients’ individual characteristics, such as different health status and lifestyle (activities, diet, etc.). These words sounded 2,500 years ago in Ancient Greece and still resonate as the definition of personalized medicine as we know it today.

## Challenges in the Implementation of Personalized Medicine and the Path Forward

In this evolving landscape, diagnostic companies have taken center stage. Boosted by easy access to molecular analysis as well as data scientists and fast computing, many thousands of studies have produced a wealth of biomarkers, algorithms, prediction models, and other clinical decision support tools to help us navigate clinical care, also in the field of transplantation.

However, the challenge lies in the implementation of these innovations in clinical practice, which is very often not happening. This relates to a lack of coordination and focus, leading to dispersion. Research in our field is often driven by local funding, with very few international, unified European, or even global programs. Furthermore, there is no consensus on the best approaches, and small sample sizes, single-centre retrospective data, and conflicts of interest can bias the development and implementation of algorithms. Many biomarkers lack independent validation and net benefit, or risk-benefit analyses are not performed. Even if validated extensively, clinical access to these tools and algorithms is hindered by regulatory requirements like the *In Vitro* Diagnostics Regulations (IVDR) in the European Union (EU) [6]. This leads to great heterogeneity in clinical practice between, for example, the EU and the United States, but also between countries and even individual centres within one country.

I am convinced that each of these hurdles to implement personalized medicine can be overcome by strong collaboration like we have observed at the ESOT Congress 2023. For instance, over the past two decades, we have clearly advanced personalized medicine for kidney transplantation [3], with much more detailed risk stratification tools with advanced immunogenetics analyses of donors and recipients and anti-HLA donor-specific antibody evaluations, which is routinely implemented in clinical practice [7, 8]; with novel non-invasive diagnostics entering clinical use [9–11]; with improved classification of rejection and biopsy-based molecular diagnostics analysis integrated in the Banff classification [12, 13], with classification of disease stage (activity vs. chronicity [12]); and with validated prognostication tools even acceptable as endpoints in drug registration trials [14, 15].

## Innovative Therapies and Biomarkers Predictive of Therapy Response

One major shortcoming is that we do not yet have predictive biomarkers that are able to predict therapy response [3]. To move our field forward and improve outcomes for our patients, we need to focus on the discovery and validation of novel therapeutic targets, test therapies that halt disease pathobiology, and find predictive biomarkers that indicate which therapies will work best in which particular patients. We can personalize care as much as we want, but if the therapeutic armamentarium sticks with toxic high-dose steroids as alpha and omega of, e.g., rejection treatment protocols, we will not improve outcomes much.

To move forward and improve patient outcomes, we need to couple the promise of personalized medicine with the extensive pipeline–outside of transplantation–of innovator drugs. This merging of personalized medicine with drug development should be our primary focus. The global immunology market is booming, and if we can attract even a fraction of it to transplantation, it can make a substantial difference for our patients.

## Socrates and the Concepts of Uncertainty

The ESOT Congress 2023 focused on realistic care, digital transformation, innovation, technology, and shared decision making. So, in essence, about how we can implement personalized medicine in our daily clinical practice. But without new drugs, this will only have marginal effects on the outcome of our patients. To move our field forward, we will need the brightest people among us to work together and move things forward.

2,500 years ago, the brightest man on Earth, according to the Oracle of Delphi, was Socrates, whose statue was used as the symbol for the ESOT Congress 2023. Socrates, a philosopher in Ancient Greece who worked and lived here on the very same ground as the congress, taught his students lessons, which are still of great value today. Socrates indicated that progress will be made through open dialogue, education, critical thinking, and most importantly, self-criticism. We indeed have to remain critical to our results and achievements. In the era of social media and self-promotion, we must embrace these principles and remain humble and critical.

Next to these principles, Socrates initiated discussions about uncertainty. He would have said: “To be uncertain is to be uncomfortable, but to be certain is to be ridiculous.” Also today, we need to embrace uncertainty. People who are very certain about themselves or their ideas or getting front stage in all aspects of society, but we observe that sometimes this is not just absurd, but also counterproductive and even dangerous.

## Uncertainty and Personalized Medicine

**FIGURE 1:**
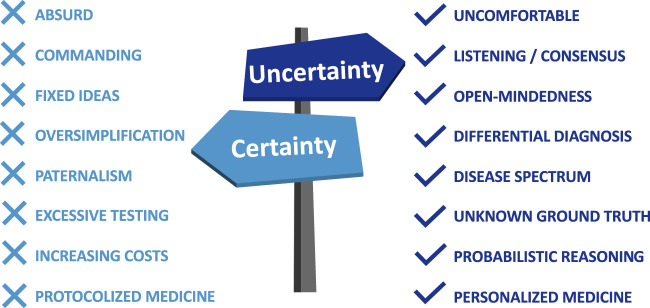
Certainty vs. uncertainty in medicine. People who are overly certain of themselves or their ideas risk to be fixed in them, and command others to follow these ideas, even if they are absurd. In medicine, certainty is also an absurdity, where diagnosis, prognosis, predicted treatment effects, etc. are usually oversimplified. It leads to paternalism towards patients and colleagues, excessive testing to find the ground truth, which can lead to rising costs. Protocolized medicine suggests such certainty to healthcare professionals, but in medicine, one size does not fit all. In contrast, uncertainty is essentially uncomfortable and vulnerable, but it this leads to a listening and consensus-driven attitude, and open-mindedness. In medicine, it is very often not possible to measure the ground truth, diseases are spectrums and overlap with other diseases, which leads to differential diagnoses. In personalized medicine, where biomarkers and clinical decision support systems inherently produce results with levels of uncertainty, probabilistic reasoning is key, both for healthcare providers and patients.

This concept of uncertainty is crucial in the implementation of personalized medicine in our clinical practice (Figure 1) [16]. A level of uncertainty is inherent to every aspect of personalized medicine, e.g., when we use risk biomarkers like donor-recipient genetic mismatch analysis or antibody evaluation, when non-invasive tests indicate a probability for ongoing disease. For disease diagnosis and disease severity, it is clear that we cannot assess the final ground truth, that we rely on consensus-based classifications like Banff, which are inherently imperfect. Prognostic algorithms for outcome prediction are available, but this is not a magic crystal ball that accurately predicts the future; there remains a lot of uncertainty in our prognostications, at the individual patient level. Treatment outcomes are often unpredictable, especially when we lack predictive biomarkers that provide information on the probability of response to a particular therapy.

Recently, it was outlined how important it becomes to embrace uncertainty in the era of clinical algorithms, but also how difficult it is to implement the thinking about uncertainty in our clinical reasoning [17]. Paraphrasing Hippocrates, no patient is just like the average patient. Many clinical decision support systems use algorithms to make predictions, in uncertain medical conditions. It is important to realize that positive and negative predictive values are very dependent on disease prevalence, which can greatly differ between populations and centres. These predictions are typically expressed as probabilities; a diagnosis becomes more or less likely, with some explicit degree of uncertainty.

These probabilistic results do not align with how most doctors typically think about whether a disease is present or absent, in a black-and-white simplistic world where certainty is readily achievable [16]. The quest for diagnostic certainty quickly leads to excessive testing, not only increasing healthcare costs but also risking false positive results and iatrogenic injury. Moreover, the principles of probabilistic clinical decision support systems perhaps clash somewhat with pathophysiology-based reasoning, which is still very relevant in the development and clinical implementation of targeted therapies, e.g., also for rejection [18].

Clinical decision making requires integration of probabilistic reasoning with acceptance of uncertainty around disease causality, because true causality usually cannot be proven in the clinical setting. I believe that Socrates would agree with this modern translation of his ideas on uncertainty. Embracing the concept that certainty is not always the end goal will be key for the future of medicine.

## The Democratic Legacy of the City-State of Athens: Consensus Needs Uncertainty

The wisdom of Ancient Greece is not only related to the thoughts of Socrates. Athens is also the city-state where 2,500 years ago, democracy, the power to the people, was invented. In the earliest years, the direct democracy in Athens was not only accessible but, in fact, obligatory for every male citizen aged 20 and above. In contrast to what was depicted by Raphael in his fresco “School of Athens” in the Vatican 2000 years later (1,509–1,511), democracy in Athens did not take place in a splendid palace. Democracy was merely an open space where the people of Athens were expected to come to listen to each other. The assembly meeting place and speaker’s platform were located near the site of ESOT Congress 2023; its ruins can still be visited today.

As is the case for personalized medicine, uncertainty is also vital for democracy. It is only when we are critical and uncertain about our own ideas and conclusions, and accept that there is not one single truth, that other people can have other ideas, that we can form consensus. We have to listen to others’ ideas, and find common ground. Otherwise, we risk to end up in toxic leadership and tyranny. Around us, we see many examples of what can happen when we give too much power to people who are too self-confident and complacent and stop listening to other opinions.

Recent examples of critical self-reflection and successful democratic processes in our field are the ESOT Transplant Learning Journey [19], the ongoing ENGAGE consensus for sensitization in transplantation [7, 8], the Banff consensus for allograft pathology [20], and the SONG-Tx initiative for defining standardized outcomes in transplant nephrology [21, 22]. Especially the latter is a good example of how important the democratic processes are for the field. Using Delphi methodology, not only health professionals, but also patients and their caregivers were able to contribute to the definition and validation of outcome measures, that will become relevant for clinical trial design.

Perhaps most importantly, the SONG-Tx initiative [21, 22] illustrates that we can access robust methods that enable to integrate patients’ perspectives in further development of the field. This allows to put the focus of research to what matters most to the patients. Explicit democratic processes enable us to integrate all opinions, also those from our main stakeholders, the patients. Such processes enable full patient centrality.

## Integrating the Concepts of Uncertainty in Patient Interactions

With such focus on what matters to patients, it is also very important to interact with the patients and their caregivers on what are the implications of personalized medicine. As described above, uncertainty is central to personalized medicine, and it will be crucial to be honest with patients about this uncertainty as well [23]. In crisis management, it is sometimes said that in times of uncertainty, honesty is the best policy. The same counts for medicine. We need to discuss together with the patients what is the impact of the new discoveries and advancements of personalized medicine, and we should not be afraid to talk openly about the uncertainties inherent to it. Not only we need to train the healthcare professionals in probabilistic thinking [17], also patients and society in general should be informed and educated about the key concepts of probabilistic reasoning in clinical decision making [23].

Only in open and honest discussions with patients as equals, away from medical paternalism, we will be able to truly individualize care. Not only must we adapt clinical approaches to individual patients’ medical conditions and the output of the biomarkers and clinical decision support algorithms, but we must also take into account less quantifiable aspects of risk appetite or aversion, expectations, quality of life, social support, and even economic considerations.

## Conclusion

In conclusion, we must learn from the wisdom of ancient Greece and the city-state of Athens, where democracy thrived, and where Socrates championed critical thinking and preaching uncertainty. The coming years, we really will need to focus on what matters most to the organ transplant recipients. Patient centricity will be key. We need much more structured concertation and collaboration, especially making the bridge between personalized medicine and innovative drug development, an important gap that is halting progress in clinical care. EU research frameworks and international funding for the transplant field are urgently necessary. Last but not least, we need different ways of communication with each other and with patients. We should embrace the democratic processes we are increasingly implementing in our community. We should allow levels of uncertainty in our discussions and train ourselves in probabilistic thinking, admitting that there is more we do not know than we know. And this very much echoes what Socrates said 2,500 years ago.
